# Implementing a 3As and ‘Opt-Out’ Tobacco Cessation Framework in an Outpatient Oncology Setting

**DOI:** 10.3390/curroncol28020115

**Published:** 2021-03-14

**Authors:** Sarah Himelfarb-Blyth, Catherine Vanderwater, Julia Hartwick

**Affiliations:** 1 Central Regional Cancer Program, Southlake Regional Health Centre, Stronach Regional Cancer Centre, Newmarket, ON L3Y 2P9, Canada; 2 Southlake Regional Health Centre, Stronach Regional Cancer Centre, Newmarket, ON L3Y 2P9, Canada; cvanderwater@southlakeregional.org (C.V.); jhartwick@southlakeregional.org (J.H.)

**Keywords:** smoking cessation, tobacco use, cancer treatment, health benefits, implementation, health care providers, quality improvement

## Abstract

Tobacco cessation has been recognized as an important goal for all ambulatory cancer centres to provide the best possible treatment outcomes and quality of life. However, cessation interventions are applied inconsistently in this setting, with less than one-half of tobacco users being offered evidence-based interventions. The ‘opt-in’ approach traditionally used in cessation, which targets patients who feel ready to quit, may limit the number of patients who are able to receive treatment, and evidence suggests that tobacco users quit at the same rate regardless of their perceived readiness. This paper reports the results of implementing a tobacco cessation framework utilizing the 3As and an ‘opt-out’ approach as a standard of cancer care at a Regional Cancer Centre. A comparison of data from 2017–2018 and 2018–2019 demonstrated an increase in the number of patients screened for tobacco use (76.9% to 90.1%, respectively), and in the number of accepted referrals to quit support (11.5% to 34.7%, respectively). The revised framework was effective at improving referral acceptance rates, despite tobacco use rates remaining stable among the two groups. This demonstrates that employing the ‘opt-out’ approach is a more effective strategy to connect patients with the smoking cessation supports required to optimize their cancer care.

## 1. Introduction

Tobacco smoking in patients with cancer has been found to cause adverse outcomes including increased treatment related toxicities, overall mortality, cause-specific mortality, and risk for second primary cancers [1]. It has been reported that quitting at the time of diagnosis can reduce the risk of dying from cancer by 30–40% [1]. In Canada, tobacco cessation has been recognized as an important goal for all ambulatory cancer centres in order to provide the best possible treatment outcomes and quality of life [2]. Oncology clinicians are able to advise patients on the benefits of quitting smoking and to encourage long-term abstinence [3]; unfortunately, tobacco cessation interventions are applied inconsistently in this setting, with less than one-half of tobacco users being offered evidence-based cessation interventions [4,5,6].

The 5As Framework (Ask, Advise, Assess, Assist, and Arrange) is a widely recognized, evidence-based intervention for tobacco cessation [7]. Within this framework a patient must report they are ready to quit and ‘opt-in’ to receive treatment. However, evidence suggests that tobacco users quit at the same rate regardless of perceived readiness and the ‘opt-in’ approach may limit the number of patients who are able to receive treatment [8]. An alternative is the ‘opt-out’ approach, where identified tobacco users are automatically referred to evidence-based cessation support [4,8]. This approach is used in a variety of healthcare settings, and its effectiveness has been demonstrated in a number of cancer sub-groups such as, lung, head & neck, and breast cancer [9,10]. 

In 2017, a revised tobacco cessation framework was released by Ontario Health-Cancer Care Ontario (OH-CCO). The adapted guidelines of this framework are grounded in 3As: Ask, Advise, and Act. The 3As are an equally effective, streamlined approach to the 5As to improve and deliver more integrated, evidence-based care [11]. The framework emphasizes an ‘opt-out’ approach for initiating referrals to cessation support services. This paper reports the results of using a tobacco cessation ‘opt-out’ model in an outpatient oncology setting. As this model becomes more established in Ontario, this study aims to provide insight into the impact of implementing an ‘opt-out’ model as a routine standard of care at a Regional Cancer Centre.

## 2. Materials and Methods

The ‘opt-out’ model was first introduced to our centre in October 2017. OH-CCO made the formal recommendation that all Regional Cancer Programs (RCP) in the province adopt this approach effective 1 April 2018. The program logistics of adopting the ‘opt-out’ model revolved around the removal of the readiness assessment components of the 5As and ‘opt-in’ model (i.e., the ‘Assess’ and ‘Assist’). Instead of assessing whether patients were ready to quit, and offering assistance if readiness was indicated, health care providers were expected to move directly from the ‘Advise’ to the ‘Act’ by initiating a referral to cessation services for every known tobacco user.

Provincial benchmarks have been set by OH-CCO for all ambulatory oncology patients to be screened for tobacco use within 28 days of their initial consultation [11]. Patients being seen for their first consultation with a Radiation or Medical Oncologist were verbally asked about their tobacco use within the previous six months by a Nurse, Oncologist, and/or Radiation Therapist during their in-person visit. If the patient did not smoke or use tobacco products, the intervention was considered complete. Tobacco users or recent quitters were subsequently advised on the benefits of quitting or remaining tobacco free in the context of their cancer therapy. Each provider was then expected to arrange a referral to appropriate cessation support services, without conducting a readiness to quit assessment. Once collected, these responses were captured in the patient’s electronic medical record (EMR) using an electronic assessment form. Within this form, the patient’s decision to ‘opt-out’ of the referral to cessation support services was documented as “declined”. The provincial guidelines also stipulate that referrals can be initiated up to 12 months after the patient’s registration [11]. This provides additional time for follow up in cases where a referral is missed or refused at the initial visit.

Three referral options, were available to patients, based on individual need. Patients could select a referral to the Smokers’ Helpline Fax Referral Program, to a community pharmacy, and/or to their primary care provider (PCP). Smokers’ Helpline is the Canadian Cancer Society’s evidence-based, established, telephone support program which offers quit planning and counselling. Patients who selected this option were referred using a fax referral form accessible within the EMR as well as by paper copy available in the clinic areas. The use of a community pharmacy for referrals was a result of a regional project which catalogued specific local Ontario Smoking Cessation Program pharmacies. These pharmacies offered specialized smoking cessation support by -trained pharmacists. Patients were presented a list of these identified, local, pharmacy partners, and a letter was faxed to a location of the patient’s choosing. If the patient chose to be referred to their PCP, a letter was faxed to that provider. Documentation outlined that a discussion about the patient’s tobacco use had taken place at the cancer centre, and that the patient had requested additional counselling and support from their PCP.

Further uptake of the ‘opt-out’ model was promoted during April 2018 to March 2019 via education sessions, coaching, audits to flag missed referrals, reminder prompts embedded in the EMR, and the introduction of a tobacco use intervention algorithm. Internal audits to flag missed referrals were implemented in April 2018 to identify patients that had not been referred to a cessation service. This report, run weekly by the clinical team, generates a list of patients with a new consult appointment in the past 20 days who did not have a referral documented despite being an identified tobacco user. Providers associated with the patient’s care are then notified of this data gap and encouraged to complete their electronic record documentation. The EMR prompts were implemented in July 2018 to help remind staff to refer and document said referrals. One prompt was a system trigger, so that when a fax referral form was selected for Smokers’ Helpline, the referral form would open on the computer as soon as the assessment form had been saved and closed. This allowed the health care provider to edit any details auto-generated in the form, and print directly from the EMR so that a copy was immediately saved in the patient’s documents. A second prompt was added to the referral component of the assessment in the EMR, which was a scripted line reading, “As part of your care today I am going to refer you to…”. This was included to remind staff to utilize open-ended language that supports the “opt-out” approach. The algorithm, released in November 2018, was created to standardize the script and the intervention steps, and made available via soft and hard copy throughout the centre. It included the clinical workflow, a reminder on available smoking cessation services, and the rationale for the adoption of the ‘opt-out’ model.

Tobacco cessation data were evaluated for the ‘opt-out’ period of April 2018 to March 2019. Data were also evaluated for the period of April 2017 to March 2018 to establish a baseline for comparison. Information collected included the number of new patients registered, the number of patients screened, the number of patients self-identified as tobacco users, the number of patients offered a referral to a quit service, and the number of patients who accepted a referral to a quit service. These data were used to calculate a performance rate (i.e., percentage) for tobacco use screening, recommending referrals, and referral acceptance. Demographic information was not directly available from OH-CCO repositories due to their analysis methodology; however, a comparable internal review of all documented cessation interventions (including new ambulatory patients, returning patients, and inpatients) was completed in order to estimate whether the control and intervention groups were comparable samples. These data were used to calculate the percentage distribution of sexes, as well as a mean and median age for each time period. Southlake Regional Health Centre’s Research Ethics Board (REB) review and approval was waived as this was considered a quality improvement initiative.

## 3. Results

An internal review of all documented cessation interventions (including new ambulatory patients, returning patients, and inpatients) found a similar sex distribution during 2017–2018 (57% Female, *n* = 2738; 43% Male, *n* = 2054) and 2018–2019 (58% Female, *n* = 2932; 42% Male, *n* = 2163). Age distribution was also similar in 2017–2018 (M = 67, Mdn = 68) compared to 2018–2019 (M = 67, Mdn = 67).

**Figure 1:**
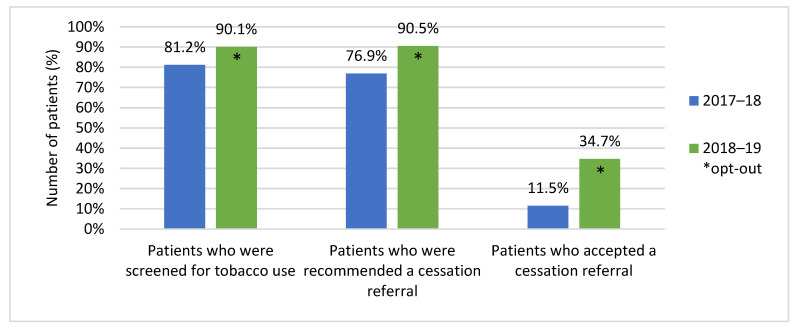
A comparison of the percentage of patients screened for tobacco use, the percentage of patients offered a referral to a quit smoking service, and the percentage of patients who accepted an offered referral in 2017/2018 and 2018/2019, respectively.

For the period April 1, 2018 to March 31, 2019, 1980 (90.1%) of 2197 patients were screened for tobacco use. Of those screened, 15.7% (*n* = 310) self-identified as tobacco users. From those identified, 90.5% (*n* = 281) were offered a referral to a smoking cessation support service, with 34.7% (*n* = 107) accepting the offered service. By comparison, for the period 1 April 2017 to 31 March 2018, 1746 (81.2%) of 2150 patients were screened for tobacco use. Of those screened, 18.4% (*n* = 322) self-identified as tobacco users, 76.9% (*n* = 248) of users were offered a referral to a smoking cessation support service, and only 11.5% (*n* = 37) of those patients accepted a referral (Figure 1). For the 2018–2019 period, internal documentation showed that 139 referrals had been made to Smokers’ Helpline, 33 patients were referred to their PCP, and 6 patients were referred to a community pharmacy of their choosing, for a total of 178 referrals. Since only 107 patients accepted referrals, a percentage of the patients who were accepting of quit support accepted a referral to more than one service (e.g., referral to PCP and Smokers’ Helpline). By comparison, for the 2017–2018 period, internal documentation showed that 61 referrals were made to Smokers’ Helpline, 12 patients were referred to their PCP, and 2 patients were referred to a community pharmacy of their choosing, for a total of 75 referrals. Since only 37 patients accepted a referral, a percentage of patients would have accepted a referral to multiple services during their intervention.

## 4. Discussion

The objective of this paper was to report on results following the implementation of a revised tobacco cessation model into routine clinical practice at a Regional Cancer Centre, from April 2018 to March 2019. Since the new model’s implementation, an increase in the number of patients screened for tobacco use was noted, suggesting that providers are successfully reaching the majority of tobacco users who may require assistance to quit. Moreover, the 3As framework emphasizing the ‘opt-out’ approach was effective at improving acceptance of cessation support, despite tobacco rates remaining stable among the two groups. While many barriers continue to exist in accessing cessation services, referring every identified user regardless of quit readiness was correlated with more patients accepting cessation support for tobacco use. In 2017–2018, only 1 in 9 known tobacco users accepted a referral to a quit service. In 2018–2019, that number improved to 1 in 3. For this reason, our data supports the ‘opt-out’ approach as a more successful method of addressing tobacco cessation in the outpatient oncology setting.

Literature on the adoption of smoking cessation frameworks in cancer care continues to emerge. One RCP published data on referral uptake using the 5As Framework which demonstrated similarly low referral acceptance rates in year 2015–2016 [12]. A second RCP reported on their program performance from 2013–2018 [13]. This report differed from our own study in two ways. Osei et al. (2019) used an in-house referral model to a smoking cessation clinic, which relied on patient readiness to drive referral acceptance. In addition, their findings focused on patient-reported outcomes and did not include the percentage of patients screened for tobacco use, offered a referral, or those who accepted quit support. Notably, neither of these studies comment on the magnitude of change in referral uptake following OH-CCO’s newly adapted smoking cessation framework. Published data most comparable to our own were from one RCP’s implementation of a digitally based smoking cessation program utilizing the 3As Framework and an electronic referral system, with modest success in referral uptake of 20% between April 2016 and March 2018 [14]. However, the screening rate using an electronic intervention system was lower than data reported in this study (62% and 90.1%, respectively), as was the referral uptake (20% and 34.7%, respectively). 

The ability to flag missed interactions where the referral may not have taken place (using internal audits), in addition to the added EMR prompts and standardized algorithm, may have helped more providers recognize referrals to cessation services as a routine part of their tobacco intervention. One of the greatest challenges when referring patients to tobacco cessation services are the limitations to accessing these supports. In Ontario, only 31% of hospitals report allocating resources to smoking cessation activities, and of these, only 42% designate staff to provide cessation support using these funds [14]. While the majority of Ontario hospitals provide no-cost nicotine replacement therapy (NRT), self-help materials, and community referrals, these processes are primarily in place for inpatient departments, and not routinely offered in the outpatient setting [15]. Making this service available to outpatients is highly dependent on resource availability at each facility and therefore, is inconsistent across the province. As a small centre, we rely on referrals to community services to support our patients to quit, thus it may be a challenge for patients to locate a service or program that meets their needs within their chosen service area.

While opportunities exist to improve our delivery of smoking cessation services, a percentage of cancer patients will continue to decline a smoking cessation referral. This is demonstrated through our data, as the acceptance rate increased from 11.5% to 34.7%, despite an increased percentage of users being offered a referral. Similarly, studies that have evaluated the effectiveness of smoking cessation interventions in patients have demonstrated that anywhere between 50% and 84.5% of patients will decline referrals to quit support [3,16]. While a familiar response from many patients is that they want to quit without formal assistance, a lower perceived risk from adverse outcomes of smoking also appears to underlie continued tobacco use. Schnoll et al. (2003) reported that cancer patients were less likely to enroll if they smoked fewer cigarettes, exhibited fewer physical symptoms, were diagnosed with a cancer type not strongly associated with tobacco use, or reported a lower stage of disease progression. These findings highlight the importance of routinely presenting tobacco interventions and smoking cessation referrals as an adjunct to cancer care, where patients already regard their providers as a trusted source of information. Offering a referral to a smoking cessation service as an essential component of care emphasizes the link between tobacco use and cancer therapy, thereby addressing the perceived weight of its role in their treatment outcomes. 

A final consideration is the impact of these referrals beyond the patient’s time at the cancer centre. Although an improvement was observed in the percentage of patients who accepted a referral, it is not possible to report on definitive patient cessation outcomes. Thus, while this review found higher volumes of accepted referrals, it is not possible to determine whether attempts to quit are temporary or sustained. Work to further improve smoking cessation activities at our centre is underway. Building patient-reported outcomes into the framework would allow providers to determine the effectiveness of their interventions on their cancer patient’s ability to stay smoke-free. Efforts are underway to expand the framework to other hospital sites and satellite locations beyond the Regional Cancer Centre. This is an important step towards a universal system in Ontario for addressing tobacco consumption in oncology.

## 5. Conclusions

The Regional Cancer Centre observed a substantial increase in the number of patients referred to a smoking cessation service through the formal adoption of an ‘opt-out’ approach, moving from 1 in 9 users accepting a referral to 1 in 3. While barriers exist in accessing cessation services, referring identified users regardless of quit readiness appears to result in a greater number of cancer patients receiving support to quit. This demonstrates that employing the ‘opt-out’ approach is a more effective strategy to connect patients with the smoking cessation supports required to optimize their cancer care. In the future, we hope to maintain our rate of accepted cessation referrals through the introduction of the framework beyond RCPs and with the possibility of patient-reported outcomes for more effective long-term follow up.

## Data Availability

Restrictions apply. For access to this data, please contact the corresponding author.
